# Cognitive impairment and the gut-brain axis during 2014–2023: a bibliometric analysis

**DOI:** 10.3389/fneur.2024.1407956

**Published:** 2024-07-05

**Authors:** Jindi He, Jiaxun Wu, Juan Liu, Hangcai Wu, Heliu Hua

**Affiliations:** ^1^Rehabilitation Department, Longyan First Affiliated Hospital of Fujian Medical University, Longyan, Fujian, China; ^2^Digestive Department, Longyan First Affiliated Hospital of Fujian Medical University, Longyan, Fujian, China

**Keywords:** cognitive impairment, gut-brain axis, bibliometrics, VOSviewer, publications

## Abstract

**Background:**

The burden on society grows as the number of individuals with cognitive impairment rises. Numerous research have discovered a connection between cognitive impairment and the gut-brain axis, which is useful in examining the pathophysiology of cognitive impairment and potential therapeutic approaches. As a result, this article explores developments and trends in the research concerning the gut-brain axis and cognitive impairment through a bibliometric analysis of the contributions made by various countries/regions, institutions, authors, and journals.

**Methods:**

We looked for articles on gut-brain axis and cognitive impairment from 2014 to 2023 in the Web of Science Core Collection. For the descriptive analysis, figures and tables were taken using GraphPad Prism 6 and WPS Office 2024. For the visual analysis of the countries/regions, institutions, authors, and keywords, VOSviewer was utilized.

**Results:**

We obtained 458 publications from 1 January 2014 to 9 September 2023. The country with the most publications (175, 38.21%) was China. The country with the greatest total number of citations (3,138, 17.22%) was the United States of America. The highest number of articles (15, 3.26%) was issued by Zhejiang University. The most published first author is Karsas M. In this field, *Nutrients* have published the most articles (24). The most often occurring keywords include “Alzheimer’s disease,” “cognitive impairment,” “gut microbiota,” “inflammation,” “diet,” etc. “Stroke,” “tau,” “probiotics,” “exercise,” “fecal microbiota transplantation,” etc. emerged later.

**Conclusion:**

An increasing amount of research has focused on the connection between cognitive impairment and the gut-brain axis. In this area, the United States of America and China have both made significant contributions. The author team’s collaboration has to be improved. Our study contributes to understanding the field’s current state and predicting its future trend.

## 1 Introduction

The typical sign of cognitive impairment is a deterioration in one or more cognitive domains, such as memory, attention, executive function, etc., either subjectively or objectively. It encompasses subjective cognitive decline, mild cognitive impairment (MCI), and dementia. According to a 2022 meta-analysis (1), 15.56% of persons 50 years of age and older who live in the community had MCI worldwide. Compared to other regions, the rates of MCI are higher in the Asia/Pacific and Latin America/Caribbean regions. Region has an impact on the prevalence of MCI. According to an analysis conducted for the global burden of disease research (2), there were 5.74 million cases of dementia in 2019, and 15.28 million cases are predicted to arise by 2050. Different countries and regions have varied growth projections.

The social and economic burden has increased due to the rise in the number of individuals suffering from cognitive impairment. In order to look into potential treatments, a lot of research has been concentrated on the pathophysiology of cognitive impairment. An increasing number of research have demonstrated a connection between the gut-brain axis and cognitive impairment. Individuals with mild cognitive impairment have diverse gut microbes. and it’s linked to executive function and attention (3). Wu et al. (4) discovered that the excretory-secretory products derived from parasites alleviate gut microbiota dysbiosis and enhance cognitive impairment caused by a high-fat diet.

Within the field of library and information science, bibliometry focuses primarily on the quantitative analysis of bibliometric data (5). Bibliometric analysis is the statistical process of analyzing published data (e.g., books, journal articles) and associated metadata (e.g., authors, abstracts, keywords) in an effort to find correlations (6). We used bibliometric analysis to identify research developments on the gut-brain axis’s association with cognitive impairment and to predict potential future trends for this relationship.

## 2 Materials and methods

### 2.1 Data sources and search strategies

**Figure 1:**
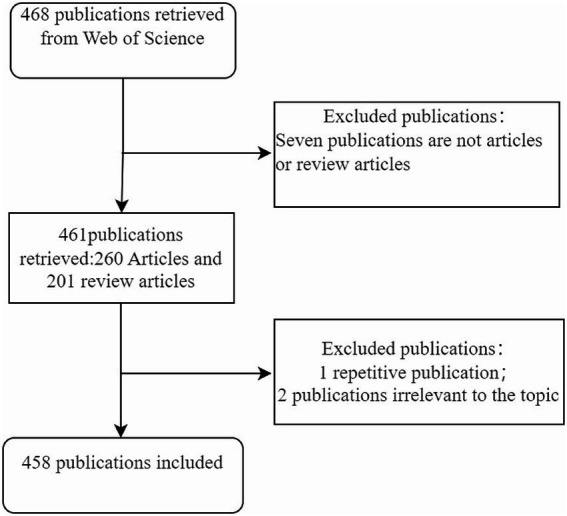
Publication screening flow chart.

The Web of Science Core Collection^1^ is widely acknowledged as one of the most commonly utilized databases for bibliometric analysis. On 10 September 2023, we conducted a search of publications in the Web of Science Core Collection. A combination of keyword searches and Medical Subject Headings (MeSH) searches were used to gather data. In the search bar, type the terms “cognitive impairment” and “gut-brain axis.” We studied a broad range of review literature to extract keywords based on the supplement of MeSH search results. The publication date ranges from 1 January 2014 to 9 September 2023. English is the language used. Table 1 presents the search queries. Only original articles and reviews were allowed in this sort of publication. Figure 1 displays the publishing screening flow chart.

**Table 1:** The search queries.

Set	Results	Search query
#3	468	#1 AND #2 AND language = English AND rate range = 2014-01-01 to 2023-09-09
#2	6186	TS = (“Axis, Brain-Gut” OR “Brain Gut Axis” OR “Gut and Brain Axis” OR “Gut-Brain Axis” OR “Axis, Gut-Brain” OR “Gut Brain Axis” OR “Brain and Gut Axis” OR “Microbiota-Gut-Brain Axis” OR “Axis, Microbiota-Gut-Brain” OR “Microbiota Gut Brain Axis” OR “Brain-Gut-Microbiome Axis” OR “Axis, Brain-Gut-Microbiome” OR “Brain Gut Microbiome Axis” OR “Microbiome-Gut-Brain Axis” OR “Axis, Microbiome-Gut-Brain” OR “Microbiome Gut Brain Axis” OR “Gut-Brain-Microbiome Axis” OR “Axis, Gut-Brain-Microbiome” OR “Gut Brain Microbiome Axis” OR “Microbiome-Brain-Gut Axis” OR “Axis, Microbiome-Brain-Gut” OR “Microbiome Brain Gut Axis” OR “Microbiota-Brain-Gut Axis” OR “Axis, Microbiota-Brain-Gut” OR “Microbiota Brain Gut Axis”)
#1	170869	TS = (“cognitive dysfunctions” OR “dysfunction, cognitive” OR “dysfunctions, cognitive” OR “cognitive impairments” OR “cognitive impairment” OR “impairment, cognitive” OR “impairments, cognitive” OR “cognitive disorder” OR “cognitive disorders” OR “disorder, cognitive” OR “disorders, cognitive” OR “mild cognitive impairment” OR “cognitive impairment, mild” OR “cognitive impairments, mild” OR “impairment, mild cognitive” OR “impairments, mild cognitive” OR “mild cognitive impairments” OR “cognitive decline” OR “cognitive declines” OR “decline, cognitive” OR “declines, cognitive” OR “mental deterioration” OR “deterioration, mental” OR “deteriorations, mental” OR “mental deteriorations”)

### 2.2 Data collection

We get all relevant publications from the database, including the following details: title, author, author address, source journals, author keywords, keyword plus, number of citations, publication date, and more.

### 2.3 Bibliometric analysis

To analyze the data, import it into WPS Office 2024, GraphPad Prism 6, and VOSviewer (1.6.19). Two components make up our analysis: 1. Descriptive analysis of publications annually, countries/regions, institutions, journals, authors, and cited references using WPS Office 2024 and GraphPad Prism 6. 2. VOSviewer is a bibliometric analysis tool that uses data from nations/regions, journals, authors, or publications to create and visualize bibliometric networks based on co-authorship, co-citation, and co-occurrence interactions (7). Use VOSviewer to carry out the analysis listed below: (1) The co-authorship analysis of the countries/regions, institutions, and authors. (2) The co-occurrence analysis of all keywords.

## 3 Results

### 3.1 Analysis of publications

**Figure 2:**
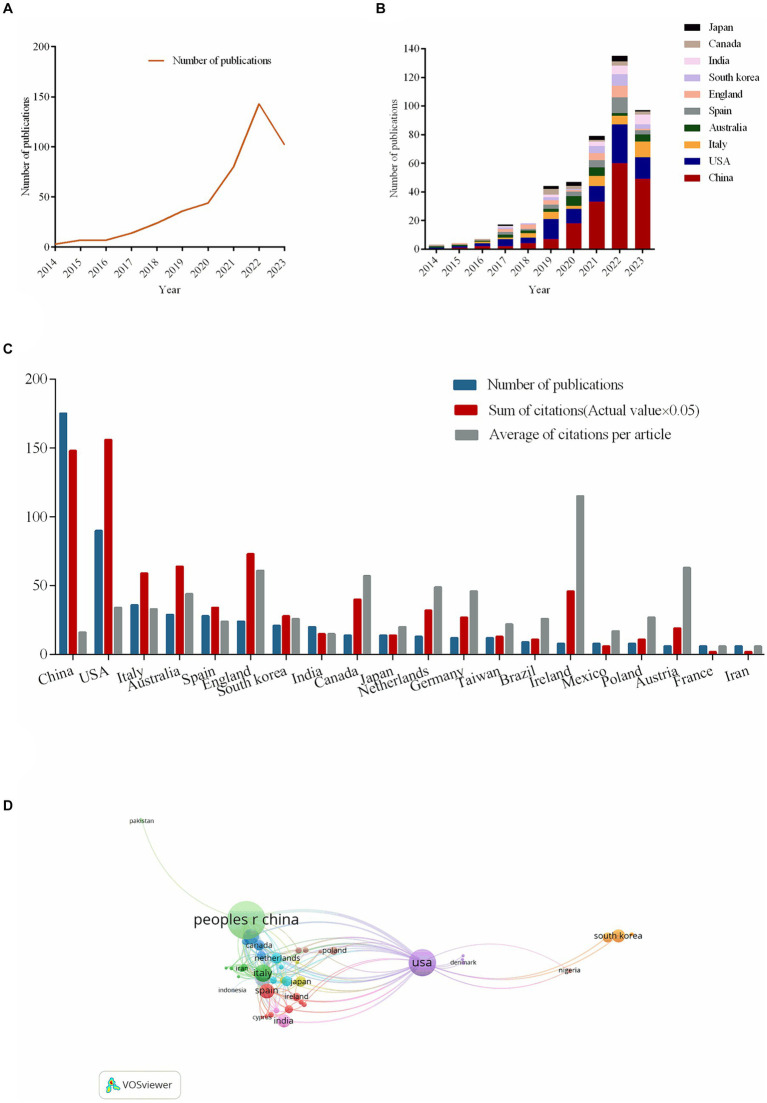
The number of publications about the topic and country/regional cooperation map. **(A)** The number of publications of the correlation between cognitive impairment and gut-brain axis from 2014 to 2023. **(B)** The number of publications of the correlation between cognitive impairment and gut-brain axis from the top 10 countries or regions per year. **(C)** The number of publications, sum of citations (×0.05) and average of citations per article in the top 20 countries or region. **(D)** The co-authorship chart between countries/regions.

After retrieving 468 publications, we chose 461 papers that fit the publishing type of original articles and reviews. Out of them, two publications are deemed irrelevant and one is repeated; ultimately, 458 papers fulfilled the study’s standards. 257 reviews make up 56.11% of all publications. Of all the articles, there are 201 original studies (43.89%), all of which were conducted *in vivo*. Of the original studies, 145 (72.14%) were conducted on animals, while 56 (27.86%) involved humans. The total number of publications indicates a growing trend from 1 January 2014 to 9 September 2023. It grew slowly prior to 2020, but it has developed quickly since then (Figure 2A).

### 3.2 Analysis of countries/regions

As Figure 2B illustrates, in 2014, research on the connection between cognitive impairment and the gut-brain axis was published only in the United States of America (USA) and Australia. Since 2015, China, Italy, Spain, England, South Africa, India, Canada, and Japan have published articles on related topics. China has published a growing number of publications every year; in 2014, there were none; by 2022, there were 60 publications (13.10%).

In terms of total publications, China ranks first (175, 38.21%), single country publications (146, 31.88%), followed by the USA and Italy (Figure 2C). All articles related to cognitive impairment and the gut-brain axis been cited 18,219 times since 2014. The USA has the highest total number of citations (3,138, 17.22%). By comparing the average number of citations per article, Ireland (115.5) ranks first. In the diagram of co-authorship between countries, the larger the node, the more the total link strength. The USA has the most cooperation with other countries, with a total link strength of 76, followed by England, with a total link strength of 51. The third, fourth, and fifth countries with strong co-authorship are China, Italy, and Spain, with a total link strength of 48, 46, and 33, respectively (Figure 2D).

China leads in both overall publications (175, 38.21%) and single country publications (146, 31.88%), with the USA and Italy following (Figure 2C). Since 2014, 18,219 times have been cited in all publications related to cognitive impairment and the gut-brain axis. With 3,138 citations (17.22%), the USA has the most citations overall. Ireland comes out on top when considering the average amount of citations per article (115.5). The greater the node’s size in the co-authorship diagram between countries/regions (Figure 2D), the stronger the total link. With a total link strength of 76, the USA leads all other countries/regions in collaboration. England comes in second with a total link strength of 51. China, Italy, and Spain, with total link strengths of 48, 46, and 33, respectively, are the third, fourth, and fifth countries with strong co-authorship.

### 3.3 Analysis of institutions

**Figure 3:**
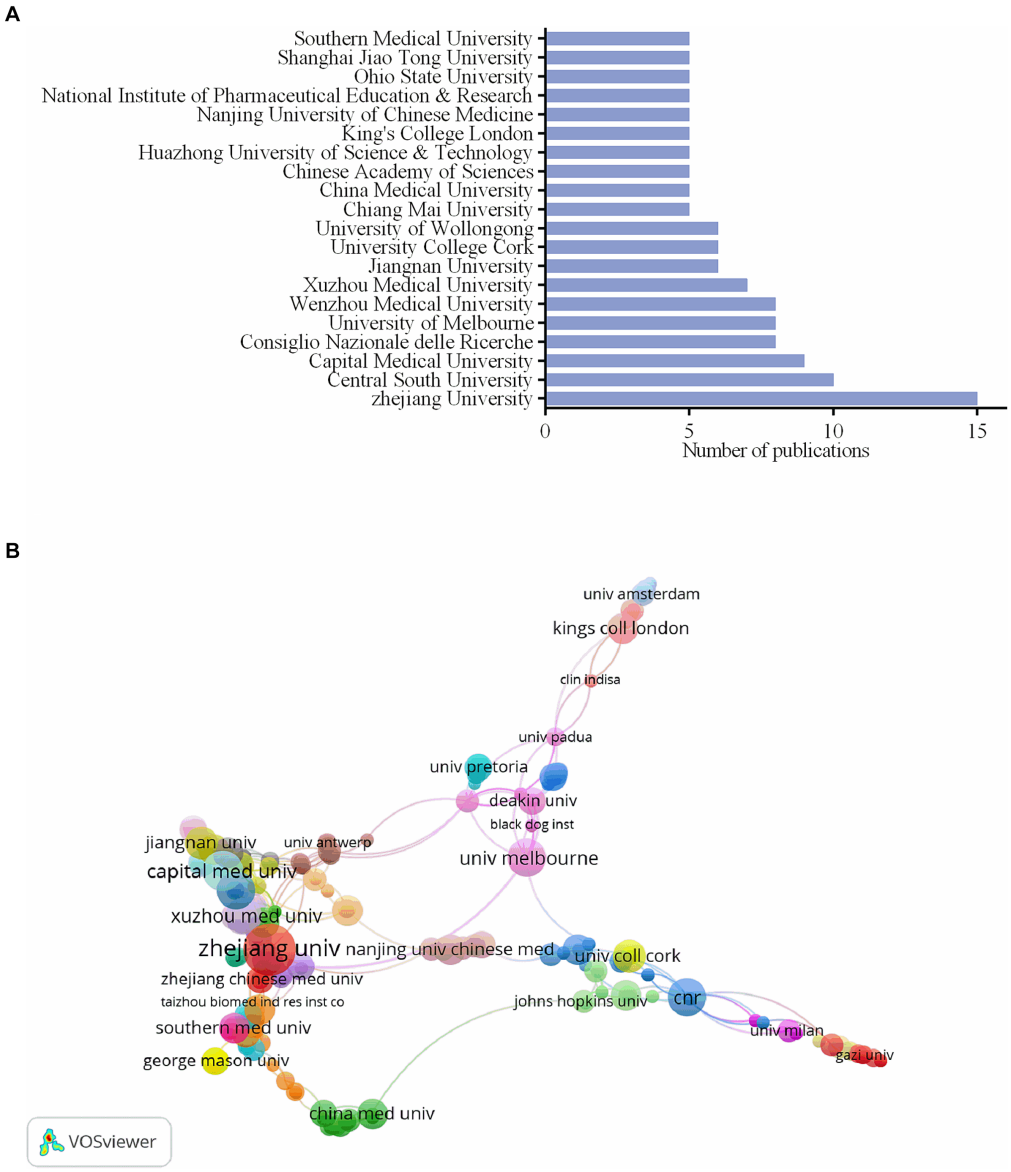
The number of publications of the top 20 institutions and institutions cooperation map. **(A)** The number of publications of the top 20 institutions. **(B)** The co-authorship chart between institutions.

Zhejiang University has the most publications, as indicated in Figure 3A, with a total of 15, or 3.26% of all publications. Central South University and Capital Medical University follow with 10, 2.17%, and 9, 1.95% of all publications, respectively. The above institutions originate in China. Twelve institutions from China, two from Australia, and six each from Italy, Ireland, Thailand, England, India, and the USA make up the top 20 institutions with the most publications. A co-authorship chart between institutions is displayed in Figure 3B. Zhejiang University in China (37), Capital Medical University in China (29), Consiglio Nazionale delle Ricerche in Italy (25), University of Pittsburgh in the USA (25), University of Naples Federico II in Italy (25), University of Melbourne in Australia (25), King’s College London in England (22), University of California Davis in the United States of America (22), Gazi University in Turkey (21), and Sun Yat-Sen University in China (21) are the top 10 institutions with the highest total link strength.

### 3.4 Analysis of authors

**Figure 4:**
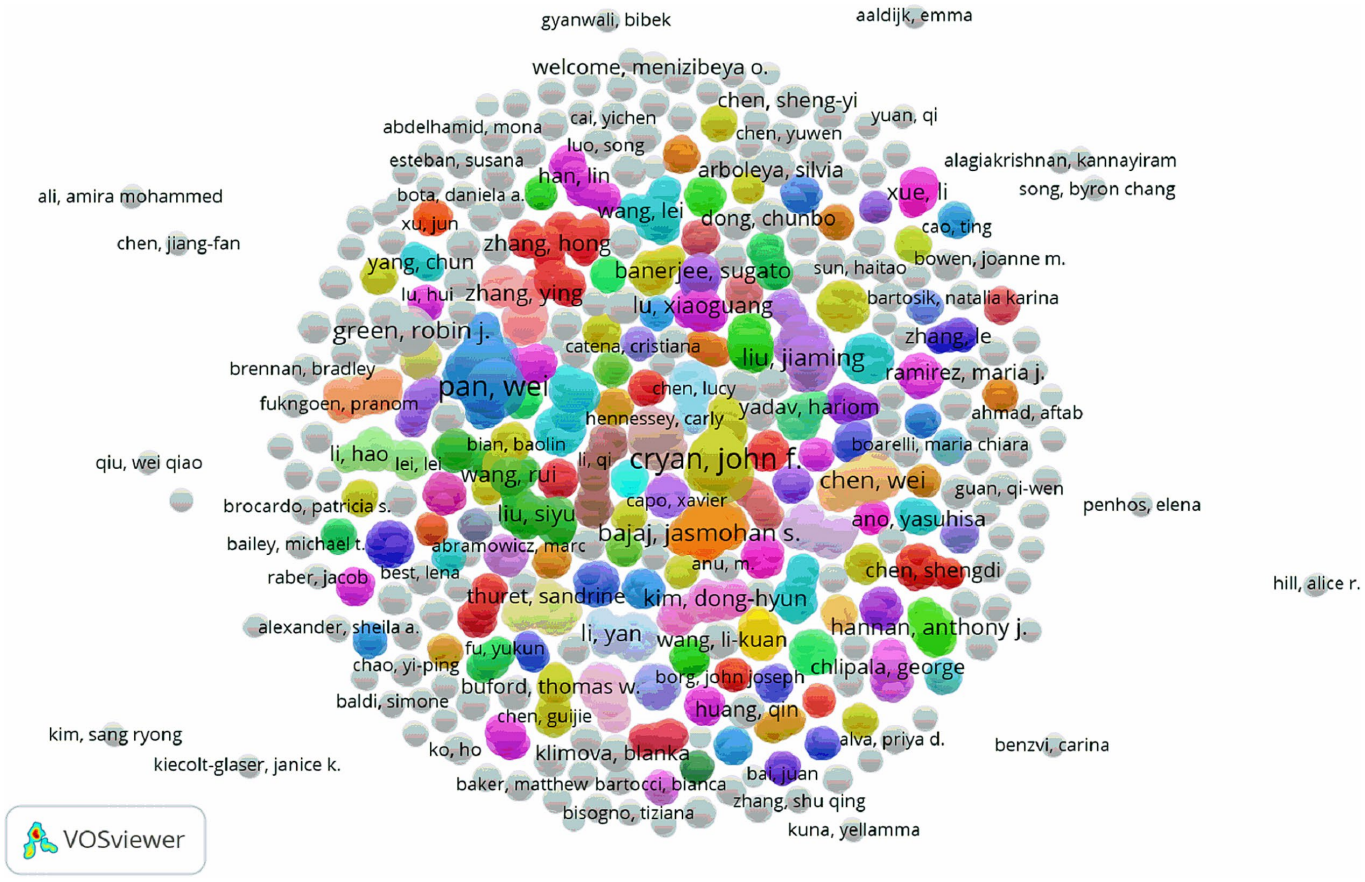
The co-authorship chart between authors.

As shown in Table 2, the top four authors published 13 papers overall with the first author, which representing 2.8% of all publications. With a total of four articles published, Karsas M from the University of Pretoria in South Africa had the most publications. Following him were three individuals who produced three publications as first authors: Bajaj JS in the USA, Zhu GS in China, and Shi HL in China. Stilling RM, an Irish author, had the highest average number of citations per article (441 times; Table 3). The co-authorship of each author is displayed in Figure 4. The authors who have the strongest overall links are Pan W (88), Yang XY (78), Shi HL (74), Yu YH (74), Bajaj JS (46), Cryan JF (44), and Gareau MG (34). While teams of authors within the same country collaborate with each other to some extent, teams within different nations do not collaborate as often.

**Table 2:** The top 4 authors with the most papers published by the first author.

Rank	First author	Country	Institution	Papers
1	Karsas M	South Africa	University of Pretoria	4
2	Bajaj JS	USA	Virginia Commonwealth University	3
2	Zhu GS	China	Jiangnan University	3
2	Shi HL	China	Xuzhou Medical University	3

**Table 3:** The top 4 authors with the highest average number of citations per paper published by the first author.

Rank	First author	Country	Institution	Papers	Citations	Average of citation
1	Stilling RM	Ireland	University College Cork	1	441	441
2	Houser MC	USA	Emory University	1	320	320
3	Liu P	China	Zhejiang University	1	262	262
4	Bonfili L	Italy	University of Camerino	1	244	244

### 3.5 Analysis of journals

**Figure 5:**
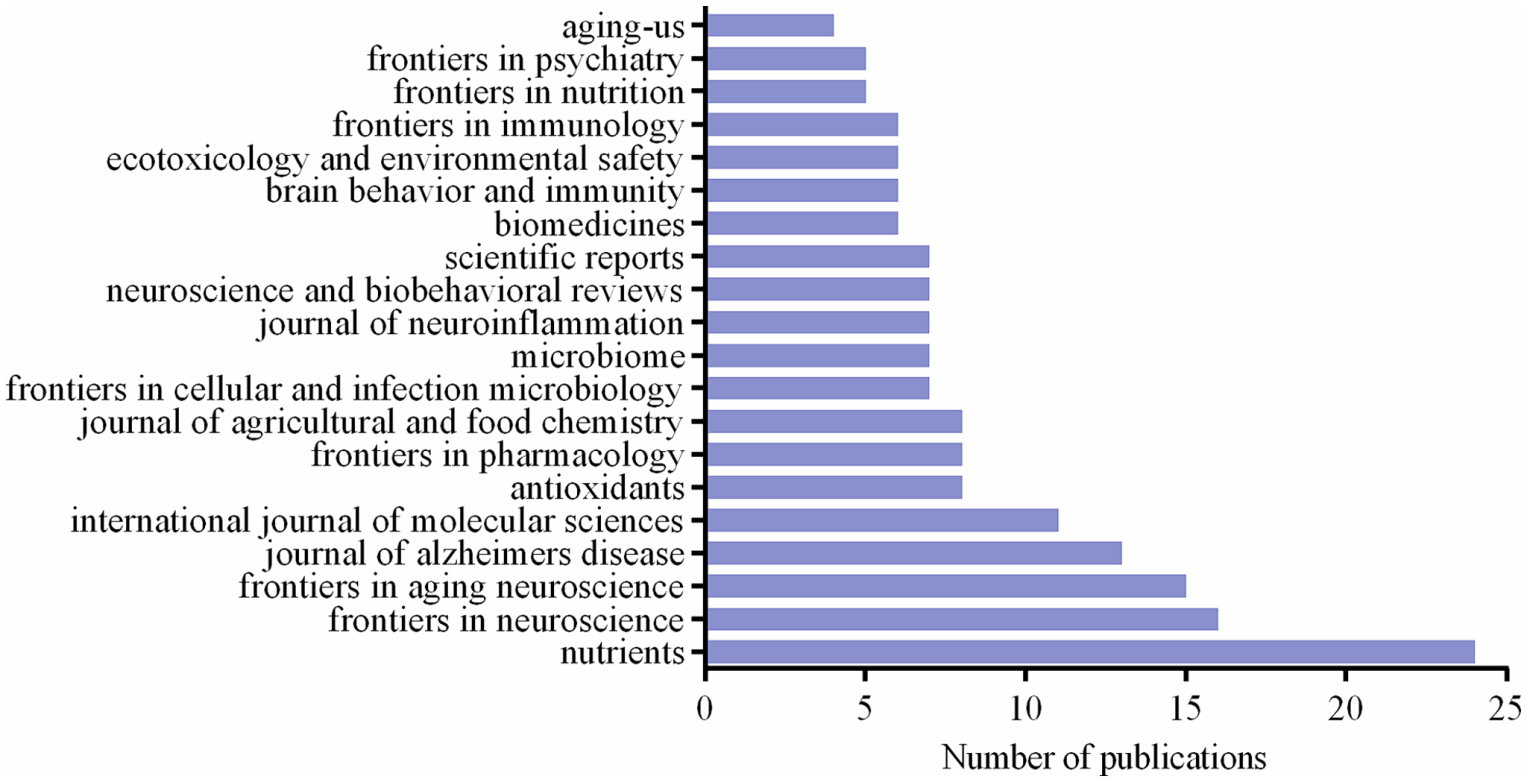
The number of publications of the top 20 journals.

A total of 176 articles, or 38.4% of the total, were published by the top 20 journals in terms of publications. Figure 5 displays the total number of articles published in the top 20 journals. With 24 articles, *Nutrients* [impact factor (IF) = 5.9,2022, Q1] has the highest number of publications, followed by 16 related publications from *Frontiers in Neuroscience*. With 15, 13, and 11 articles published, *Frontiers in Aging Neuroscience*, *Journal of Alzheimer’s Disease*, and *International Journal of Molecular Sciences* were the top 3, 4, and 5 journals, respectively. Microbiome, with 7 publications, had the greatest IF of these 20 journals (IF = 15.5, 2022, Q1).

### 3.6 Analysis of cited references

Table 4 lists the top 10 papers that have been cited the most. There were at least 181 citations for each of these papers. The paper titled “The neuropharmacology of butyrate: The bread and butter of the microbiota-gut-brain axis?” by Stilling RM et al., published on *Neurochemistry International* in 2016, received 441 citations overall, making it the most cited paper annually on average (55.13 times). The period covered by these 10 publications was from 2016 to 2020. *Alzheimer’s & Dementia*, *Brain Behavior and Immunity*, and *Movement Disorders* produced three highly referenced articles in 2019.

**Table 4:** The top 10 papers cited most frequently related to cognitive impairment and gut-brain axis.

Rank	Title	Corresponding author	Source Title	Publication year	Citations	Average citations per year
1	The neuropharmacology of butyrate: The bread and butter of the microbiota-gut-brain axis?	Stilling RM; Cryan JF	Neurochemistry International	2016	441	55.13
2	The gut-brain axis: is intestinal inflammation a silent driver of Parkinson’s disease pathogenesis?	Tansey MG	NPJ Parkinsons Disease	2017	320	45.71
3	Altered bile acid profile associates with cognitive impairment in Alzheimer’s disease-An emerging role for gut microbiome	Kaddurah-Daouk R; Kastenmuller G; Saykin AJ	Alzheimers and Dementia	2019	275	55
4	Altered microbiomes distinguish Alzheimer’s disease from amnestic mild cognitive impairment and health in a Chinese cohort	Wang BH	Brain Behavior and Immunity	2019	262	52.4
5	Microbiota modulation counteracts Alzheimer’s disease progression influencing neuronal proteolysis and gut hormones plasma levels	Bonfili L	Scientific Reports	2017	244	34.86
6	Microbiome-host systems interactions: protective effects of propionate upon the blood–brain barrier	Hoyles L; McArthur S	Microbiome	2018	236	39.33
7	Gut microbes and metabolites as modulators of blood–brain barrier integrity and brain health	Carding SR	Gut Microbes	2020	213	53.25
8	The gut microbiota-derived metabolite trimethylamine N-oxide is elevated in Alzheimer’s disease	Bendlin BB; Rey FE	Alzheimers Research and Therapy	2018	210	35
9	Alzheimer’s disease and gut microbiota	Bisogno T; Piscitelli F	Science China-Life Sciences	2016	198	24.75
10	Unraveling gut microbiota in Parkinson’s disease and atypical parkinsonism	Cereda E	Movement Disorders	2019	181	36.2

### 3.7 Analysis of keywords

**Figure 6:**
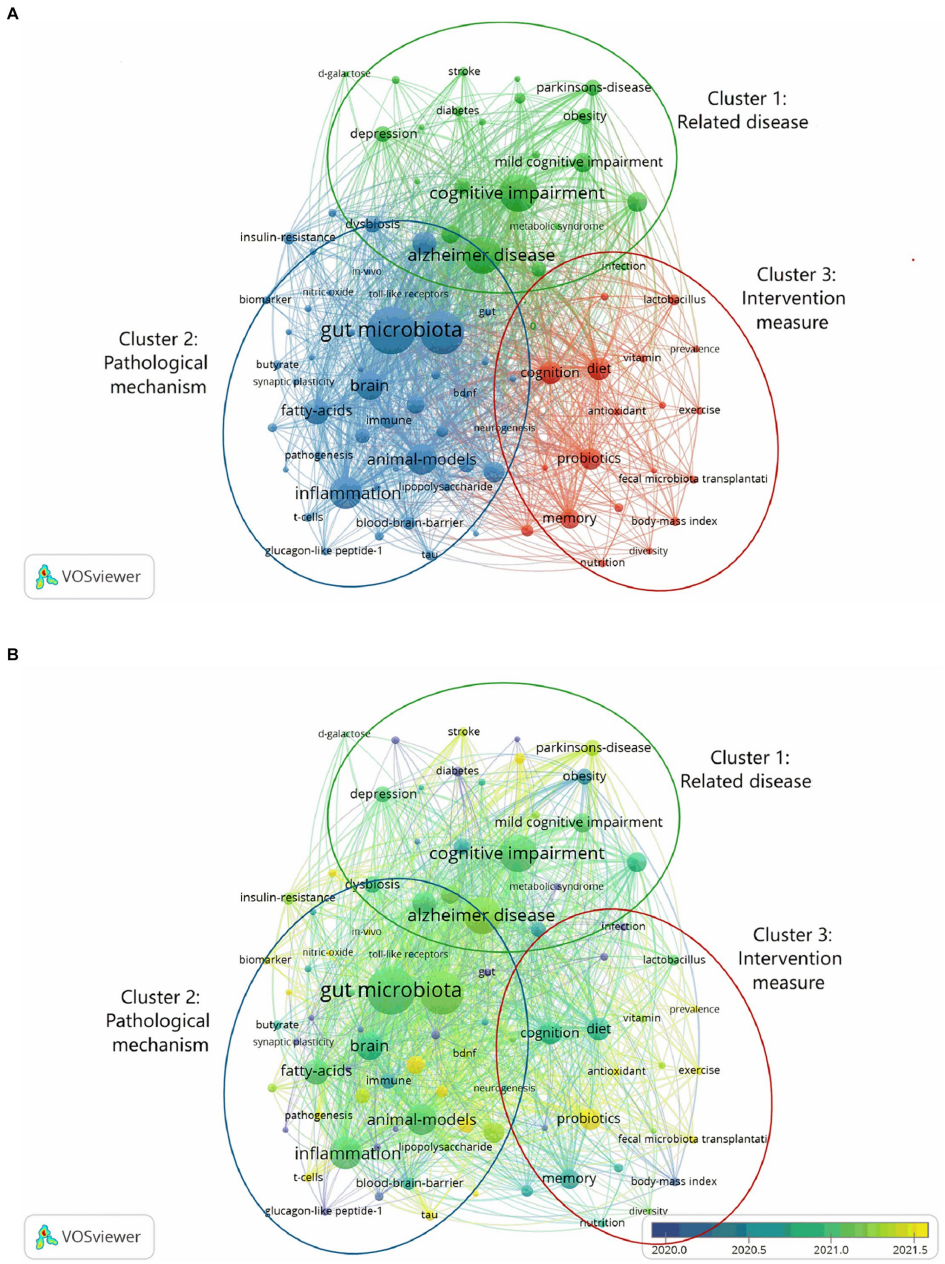
Keyword co-occurrence chart. **(A)** Different colors represent different clusters. All keywords were divided into 3 clusters. **(B)** The average occurrence year of keywords is different and the color is different. The closer the blue is, the earlier the keywords appear, and the closer the yellow is, the later the keywords appear.

We analyzed all of the keywords that were taken out of the publications using VOSviewer. As seen in Figure 6A, keywords are terms found in the author keywords and keyword plus of all articles; terms found more than six times are used to create a visual map. The frequency of keywords appears increases with node size. Three clusters were created out of the keywords: related disease (cluster 1), pathological mechanism (cluster 2), and intervention measure (cluster 3). Within the related disease cluster, the most often occurring keywords were: “cognitive impairment” (195 times), “Alzheimer’s disease” (196 times), “dementia” (57 times), and “aging” (49 times). Within the cluster of pathological mechanism, the following keywords are most often mentioned: “gut microbiota” (322 times), “gut-brain axis” (259 times), “inflammation” (151 times), “fatty acids” (89 times), and “amyloid-beta” (76 times). “Cognition” (74 times), “diet” (71 times), and “probiotics” (66 times) were the most commonly occurring cluster words in the intervention measure. The color of VOSviewer is determined by the average appearing year (AAY) of the keywords, as illustrated in Figure 6B. The sooner the keyword occurs, the closer the blue represents, and the later the keyword appears, the closer the yellow represents. “Irritable bowel syndrome” (AAY = 2018) emerged earlier in the cluster 1, whereas “stroke” (AAY = 2021) occurred later. In cluster 2, tau’s AAY was 2022 while glucagon-like peptide-1’s AAY was 2018. “Neuroprotection” (AAY = 2019) emerged first in cluster 3, while “probiotics” (AAY = 2021), “fecal microbiota transplantation” (AAY = 2021), and “exercise” (AAY = 2021) emerged later.

## 4 Discussion

We are paying more and more attention to cognitive impairment as its incidence rises around the world. Reducing modifiable risk factors and deepening research into the biological basis of cognitive impairment are predicted to lower the prevalence of cognitive impairment. There is evidence from numerous studies suggesting the gut-brain axis and cognitive impairment are related, and during the past 10 years, there has been a gradual increase in this field of study. Research hotspots and future trends in this field are examined through a bibliometric analysis of the relationship between cognitive impairment and the gut-brain axis.

It is noteworthy that various countries or regions have varying rates of cognitive impairment prevalence. A higher percentage of people with mild cognitive impairment live in East Asia/Pacific or Latin America/Caribbean (1). These regions include China, Japan, Mexico, Brazil, and the USA. While there was an increase in dementia cases in every country, the regions with the greatest increases in terms of percentage change were North Africa, the Middle East, and eastern sub-Saharan Africa (2). The rising prevalence and regional differences have prompted these regions to invest in research on the relationship between cognitive impairment and the gut-brain axis. China is the country with the greatest production in this field, as evidenced by the number of articles it has published about the connection between cognitive impairment and the gut-brain axis. However, China is not as influential as Ireland in this field and its articles are not as frequently cited. Prior to other nations, the USA focused on the connection between cognitive impairment and the gut-brain axis, and the number of studies grew nearly yearly. Afterwards, significant contributions in this area have also been made by China, Italy, Australia, Spain, and England. It was discovered that the USA and England work closely with other nations in the co-authorship among countries/regions. Additionally, China, Italy, and Spain frequently collaborate with other nations.

China has the institutions that have conducted the most study on the connection between cognitive impairment and the gut-brain axis. China’s Zhejiang University, Capital Medical University, and Central South University have all made significant advances in this area. Institutions in close collaboration with other institutions include Zhejiang University in China, Capital Medical University in China, Consiglio Nazionale delle Ricerche in Italy, University of Pittsburgh in the USA, University of Naples Federico II in Italy, and University of Melbourne in Australia. Collaboration promotes the furtherance of research, regardless of whether it is within institutions or between nations.

The most productive author, Karsas M. from South Africa, published the most articles with the first author, and the most influential author, Stilling RM. from Ireland, published the most cited papers with the first author. The lack of collaboration throughout the author teams could be attributed to different research directions or geographic restrictions, thus more has to be done to foster increased teamwork, foster inter-team learning, and foster in-depth research.

*Nutrients* is the most productive journal on the relationship between cognitive impairment and the gut-brain axis. It focuses mostly on dietetics and nutrition research. This is because the intervention of the gut-brain axis involves dietary factors. Three of the top five journals are on neurology, which are *Frontiers in Neuroscience*, *Frontiers in Aging Neuroscience* and *Journal of Alzheimers Disease*. This is due to the tight connection between disorders of the neurological system and cognitive decline. *Microbiome*, a journal that focuses mostly on studying the microbial community, has the greatest IF out of the top 20 journals.

Higher influence in this field is associated with more cited papers. “The neuropharmacology of butyrate: The bread and butter of the microbiota-gut-brain axis?” was the most frequently cited article. This study examines how butyrate, a byproduct of gut microbes, affects several facets of human physiology, including the brain and behavior. The research of the gut microbiome’s involvement in neurological disorders linked to cognitive impairment was the subject of the second, third, fourth, eighth, ninth, and tenth most cited papers. The recently released papers should be carefully examined because they are not frequently cited.

The three clusters in the co-occurrence analysis graph of keywords are associated disease, pathological mechanism, and intervention measure. The frequency of occurrence of keywords indicates their concern in the field of the relationship between cognitive impairment and gut-brain axis. In Figure 6A, the focus of intervention measure is relatively small, and the resources invested are relatively small. It is necessary to increase the research on treatment options and look forward to more effective treatment measures. The keywords of pathological mechanism have been published recently, which may be related to the transfer of research in China and the USA to this aspect in the past 10 years. In addition, it may be because researchers have a strong interest in the relationship between intestinal brain axis and cognitive impairment. There are many factors involved in the nervous system, endocrine system, immune system and so on (8). This requires researchers to continue to dig deep and explore deep mechanisms to provide a basis for subsequent treatment.

In the cluster of related disease, Alzheimer’s disease appears the most frequently and is the focus of the study. A neurodegenerative condition, Alzheimer’s disease gradually advances from MCI to severe dementia. According to studies (9, 10) on humans and animals, Alzheimer’s disease may be influenced by the gut-brain axis. The aging population has led to a major increase in attention being paid to age-related cognitive decline. According to research (11), changes in the gut microbiota, increased intestinal permeability, and inflammation may all play significant roles in the development of age-related cognitive decline. In recent years, stroke has been a prominent topic for research. Stroke has gained a lot of attention and is currently a popular study issue. Hammond et al. (12) discovered that butyrate-producing microbiome, secondary bile acid-producing taxa, and equol-producing taxa were much lower in stroke patients, and that modifications to these microbiotas were linked to cognitive impairment following stroke. In addition to neurological diseases, there are other diseases that cause cognitive impairment, such as obesity. Studies have shown that high-fat diet-induced cognitive impairment in mice is associated with intestinal Janus kinase-3 deficiency (13).

Gut microbiota is a major focus in the pathogenic mechanism clustering. Numerous microbial communities, such as bacteria, fungus, and viruses, inhabit our skin, gastrointestinal system, and other tissues. The human intestine contains more than 100 trillion bacteria (14). Eleven distinct phyla make up the human gut microbiota, the most common of which are Firmicutes, Proteobacteria, Bacteroidetes, and Actinobacteria (15). Changes in the composition of the gut microbiota can be brought about by illness or aging. For example, probiotic bifidobacteria strains have been shown to decrease with age (16). The gut-brain axis facilitates bidirectional communication between the gut and the central nervous system, controlling the immunological, neurological, neuroendocrine, and metabolic systems (8). Key metabolites of the gut microbiota are short-chain fatty acids (17), and a decrease in these fatty acids is linked to a decline in cognitive function (18). Systemic inflammation may result from dysbiosis of the gut microbiota. Pathological characteristics of Alzheimer’s disease include neuroinflammation (19). By identifying pathogens from microbial sources, toll-like receptors start the inflammatory process. Microglia from Alzheimer’s patients have been shown to exhibit toll-like receptors, suggesting a connection between inflammation and the onset of Alzheimer’s disease (20). Toll-like receptor signaling pathways-induced neuroinflammation is linked to age-related cognitive impairment (21). One of the pathological characteristics of Alzheimer’s disease is the buildup of extracellular amyloid-beta in the brain (19, 22). According to research by Sun et al. (22), amyloid-beta deposition may transfer from the gastrointestinal system to the brain and cause cognitive impairment. There has been a shift in research focus toward tau in recent times. One of the pathological characteristics of Alzheimer’s disease is cellular phosphorylation tau tangles (19). The gut microbiota of patients with Alzheimer’s disease-related cognitive impairment is linked to amyloid protein cascade markers like tau plasma phosphorylation (23).

The third cluster intervention measure focuses mostly on diet and cognition. By decreasing inflammation via the gut-brain axis, diet can halt the deterioration of cognition (24, 25). Certain diets, such high-fat diets, can lead to obesity-related cognitive impairment, therefore not all diets are good for you (13). Recent research has shown that supplemental probiotics, fecal microbiota transplantation, and exercise are all hot subjects. Probiotics have been shown in animal studies to modify the gut microbiota, reduce inflammation caused by lipopolysaccharides, change the concentration of neurotransmitters, and enhance cognitive performance (26, 27). A study conducted by Park et al. (28), discovered that fecal microbiota transplantation could postpone dementia patients’ cognitive impairment. Fecal microbiota transplantation has been shown to decrease neuroinflammation and enhance cognitive function in animal trials (29). Exercise can increase the diversity of gut bacteria, decrease intestinal inflammation, decrease intestinal inflammation, improve cognitive function, and alleviate fatigue (30).

There are intersections between these three keyword clusters. Related diseases like Alzheimer’s, stroke, and other neurological conditions, along with aging, obesity, and other factors, all contribute to cognitive impairment. The gut brain axis acts as a bridge between the dysfunction of intestinal flora and the loss in cognitive function that results from these disorders. The pathogenic mechanism includes oxidative stress, neuroinflammation, and other factors. Currently, intestinal flora imbalance can be adjusted and inflammation can be decreased through diet, probiotic supplementation, and other therapy regimens.

## 5 Limitations

This bibliometric analysis, which is based on VOSviewer, examines the macro link between the gut-brain axis and cognitive decline. However, the finer points, such as the pathophysiological connection between the gut-brain axis and cognition impairment in various disorders, have not been thoroughly examined. Articles published after 9 September 2023, were not included in the analysis.

## 6 Conclusion

Our bibliometric analysis of the correlation between the gut-brain axis and cognitive impairment revealed an annual rise in research in this area. Greater output in this field is produced in China and the USA. Universities make up the majority of the institutions with the most publications. It is necessary to improve the collaboration between the author’s teams. Focused keywords include Alzheimer’s disease, gut microbiota, and inflammation, etc. Research hotspots include probiotics, fecal microbiota transplantation, exercise, and more. Our research contributes to our understanding of the current state of the field on the relationship between cognitive impairment and the gut-brain axis and forecasts the direction of future studies in this area.

## Data availability statement

The datasets presented in this study can be found in online repositories. The names of the repository/repositories and accession number(s) can be found in the article/supplementary material.

## Author contributions

JH: Writing – original draft, Writing – review & editing. JW: Formal analysis, Methodology, Visualization, Writing – review & editing. JL: Data curation, Writing – review & editing. HW: Data curation, Writing – review & editing. HH: Supervision, Writing – review & editing.

## Footnotes

^1^https://www.webofscience.com/wos/woscc/basic-search
